# Serum S100B and Suicidal Ideation in Major Depressive Disorder: Evidence for a Trauma-Mediated Neurobiological Pathway

**DOI:** 10.3390/ijms27114736

**Published:** 2026-05-25

**Authors:** Celal Yaşamalı, Şengül Kocamer Şahin, Bahadır Demir, Gülçin Elboğa, Abdurrahman Altındağ

**Affiliations:** 1 Department of Psychiatry, Gaziantep City Hospital, 27090 Gaziantep, Turkey; 2 Department of Psychiatry, Faculty of Medicine, Gaziantep University, 27310 Gaziantep, Turkey; snglkcmr@hotmail.com (Ş.K.Ş.); drbahadirdemir@gmail.com (B.D.); gulcincinpolat@yahoo.com (G.E.); draltindag@yahoo.com (A.A.)

**Keywords:** S100B, major depressive disorder, suicidal ideation, childhood trauma, mediation analysis

## Abstract

Serum S100B has been proposed as a peripheral biomarker associated with neuroinflammatory and astroglial stress-related processes in major depressive disorder (MDD). This study aimed to evaluate serum S100B levels in patients with MDD and suicidal ideation and to investigate whether childhood trauma mediates the relationship between suicide probability and S100B levels. This study included patients with MDD and suicidal ideation (*n* = 29), patients with MDD without suicidal ideation (*n* = 30), and healthy controls (*n* = 29). Serum S100B levels were measured before and after treatment in patients with suicidal ideation. Suicide Probability Scale (SPS), Childhood Trauma Questionnaire (CTQ), and Rosenberg Self-Esteem Scale (RSES) scores were assessed. Group comparisons were performed using Mann–Whitney U and Kruskal–Wallis tests with Dunn–Bonferroni post hoc analysis. Logistic regression and mediation analyses were conducted to examine the relationships among suicide probability, childhood trauma, and S100B levels. Pre-treatment serum S100B levels were significantly higher in patients with MDD and suicidal ideation compared with healthy controls (median 10.95 vs. 8.97 pg/mL, *p* = 0.001), whereas post-treatment levels did not differ between groups (median 7.84 vs. 8.97 pg/mL, *p* = 0.323). Within-group analysis demonstrated a significant reduction in S100B levels after treatment (*Z* = −3.359, *p* < 0.001). Additional three-group comparison revealed a significant overall difference in S100B levels among the study groups (*H* = 8.17, *p* = 0.017). Logistic regression analysis showed that serum S100B levels were independently associated with suicidal ideation (*OR* = 1.14, 95% *CI* 1.02–1.27, *p* = 0.021). Mediation analyses demonstrated a significant indirect effect of suicide probability on S100B levels through childhood trauma (Sobel *Z* = −2.45, *p* = 0.014). Serum S100B levels were elevated during the acute phase of MDD with suicidal ideation and decreased following treatment; however, the specificity of this longitudinal change to suicidality could not be determined within the present study design. The relationship between suicide probability and S100B levels appears to be mediated by childhood trauma, suggesting that S100B may reflect trauma-related neurobiological vulnerability rather than a disease-specific biomarker of suicidality. These findings support a potential association between peripheral glial-related biomarkers and stress-responsive neurobiological processes underlying suicidality.

## 1. Introduction

Suicide remains a major global public health concern, accounting for approximately 800,000 deaths worldwide each year and representing one of the leading causes of mortality among adolescents and young adults [1]. Suicidal behavior encompasses a complex spectrum ranging from suicidal ideation to suicide attempts and completed suicide, and its prediction continues to challenge clinical psychiatry due to its multifactorial and dynamic nature. Although suicidal behavior may occur across a wide range of psychiatric and non-psychiatric conditions, mood disorders—particularly major depressive disorder (MDD)—constitute the most prominent diagnostic category associated with suicide risk [2].

Epidemiological studies indicate that nearly 90% of individuals who attempt or die by suicide have at least one diagnosable psychiatric disorder, with MDD being the most prevalent [3]. Nevertheless, depression alone lacks sufficient specificity to explain suicidal behavior, as only a subset of patients with MDD develop suicidal ideation or engage in suicide attempts [4]. This observation underscores the need to identify biological and psychosocial factors that modulate suicide risk within depressive populations.

Among psychosocial determinants, childhood trauma has emerged as one of the most robust and consistently replicated risk factors for suicidal behavior [5]. Exposure to physical, emotional, or sexual abuse and neglect during early life has been shown to induce long-lasting alterations in stress-response systems, particularly the hypothalamic–pituitary–adrenal (HPA) axis, thereby increasing vulnerability to affective disorders and suicidal behavior in adulthood [6,7]. In parallel, childhood trauma is strongly associated with reduced self-esteem, maladaptive cognitive schemas, and impaired emotion regulation, all of which may contribute to heightened suicide risk in individuals with MDD [8]. Despite extensive evidence supporting these associations, the biological pathways through which early-life adversity translates into increased suicidality remain incompletely understood.

In recent years, increasing attention has been directed toward neurobiological markers that may reflect glial dysfunction, neuroinflammation, or blood–brain barrier (BBB) integrity in psychiatric disorders. S100B is a calcium-binding protein predominantly expressed by astrocytes and Schwann cells in the central nervous system [9,10]. Under physiological conditions, S100B is present at low concentrations in peripheral blood; however, elevated serum levels have been reported in various neurological and psychiatric conditions [11,12,13]. Experimental and clinical studies suggest that S100B plays a role in neurodevelopment, synaptic plasticity, glial activation, and intracellular signaling, while excessive extracellular concentrations may exert neurotoxic or proinflammatory effects [14,15,16].

Importantly, serum S100B has been proposed as an indirect marker of BBB permeability rather than direct neuronal injury, particularly in the absence of overt neurological disease [14,17,18]. This characteristic renders S100B a biologically plausible candidate biomarker linking psychosocial stress, neuroinflammatory processes, and psychiatric symptomatology. Several studies have demonstrated significantly elevated serum or cerebrospinal fluid S100B levels in patients with mood disorders, including MDD, compared with healthy controls [19,20,21,22,23]. Moreover, antidepressant treatment has been associated with normalization or S100B reduction, which may reflect biological changes associated with clinical improvement [24].

Beyond depression, emerging evidence implicates S100B in suicidality. Postmortem and clinical studies have reported higher S100B concentrations in individuals who died by suicide or exhibited severe suicidal ideation, independent of specific psychiatric diagnoses [21,25,26]. Notably, Falcone et al. demonstrated that elevated S100B levels were associated with both suicidal ideation and a history of childhood trauma, raising the possibility that early-life stress may influence suicide risk through glial or BBB-related mechanisms reflected by S100B alterations [27].

Although previous studies have reported associations among S100B levels, suicidality, and childhood trauma, important gaps remain in the literature. Most available studies have relied on cross-sectional designs, and longitudinal changes in S100B levels during treatment of MDD with suicidal ideation remain insufficiently investigated. In addition, the potential indirect relationships among childhood trauma, suicide probability, and S100B levels have not been comprehensively evaluated within an integrated analytical framework. Addressing these gaps may contribute to a better understanding of stress-related neurobiological processes associated with suicidality in major depressive disorder. Therefore, the present study aimed to investigate both longitudinal changes in serum S100B levels and the potential indirect associations among suicide probability, childhood trauma, and peripheral S100B concentrations in patients with major depressive disorder.

## 2. Results

Sociodemographic and baseline clinical characteristics of the three study groups are presented in Table 1. There were no statistically significant differences among patients with MDD and suicidal ideation, patients with MDD without suicidal ideation, and healthy controls in terms of gender distribution (*p* = 0.801), mean age (*p* = 0.874), years of education (*p* = 0.691), or marital status distribution (*p* = 0.845). Baseline HDRS scores were significantly higher in patients with suicidal ideation compared with patients without suicidal ideation (28.4 ± 5.2 vs. 22.1 ± 4.8, *p* < 0.001) (Table 1).

Comparison of serum S100B levels between patients with MDD and suicidal ideation and healthy controls before and after treatment is shown in Table 2. In the pre-treatment period, median serum S100B levels were 10.95 pg/mL (Q1–Q3: 9.08–28.95) in the patient group and 8.97 pg/mL (Q1–Q3: 7.11–10.00) in the control group, with a statistically significant difference between the groups (*p* = 0.001). In the post-treatment period, median serum S100B levels were 7.84 pg/mL (Q1–Q3: 6.49–9.81) in patients and 8.97 pg/mL (Q1–Q3: 7.11–10.00) in controls, and no statistically significant difference was observed between the groups (*p* = 0.323). Within-group analysis in the patient group demonstrated a significant difference between pre-treatment and post-treatment serum S100B levels (Z = −3.359, *p* < 0.001) (Table 2).

Baseline serum S100B levels from patients with MDD without suicidal ideation were included in the three-group comparison analysis. Comparison of serum S100B levels across the three study groups is shown in Table 3. In order to explore whether serum S100B levels differed across all study groups, an additional analysis including patients with MDD with suicidal ideation, patients with MDD without suicidal ideation, and healthy controls was performed using the Kruskal–Wallis test. The analysis revealed a significant overall difference among the groups (H = 8.17, *p* = 0.017) (Table 3). Post hoc Dunn–Bonferroni comparisons indicated that serum S100B levels were significantly higher in patients with MDD with suicidal ideation compared with healthy controls (*p* = 0.012) and patients with MDD without suicidal ideation (*p* = 0.041), whereas no significant difference was observed between patients with MDD without suicidal ideation and healthy controls (*p* = 0.284) (Table 3).

Comparison of clinical scale scores between patients with MDD with and without suicidal ideation is shown in Table 4. The mean Suicide Probability Scale score was 87.45 ± 11.14 in patients with suicidal ideation and 46.20 ± 17.05 in patients without suicidal ideation, and the difference between the groups was statistically significant (*p* = 0.001). The mean Rosenberg Self-Esteem Scale score was 14.28 ± 5.71 in the suicidal ideation group and 13.76 ± 5.34 in the non-suicidal ideation group, with no statistically significant difference between the groups (*p* = 0.725). The mean Childhood Trauma Questionnaire score was 45.17 ± 14.76 in patients with suicidal ideation and 30.50 ± 12.41 in those without suicidal ideation, showing a statistically significant difference between the groups (*p* = 0.001) (Table 4).

Logistic regression analysis was conducted using suicidal ideation status as the dependent variable. Serum S100B level was entered as the primary independent variable, while childhood trauma, self-esteem, age, and sex were included as covariates. Logistic regression analysis predicting suicidal ideation is shown in Table 5. Logistic regression analysis was conducted to evaluate whether serum S100B levels independently predicted suicidal ideation among patients with major depressive disorder. In the multivariable model including childhood trauma, self-esteem, age, and sex, serum S100B levels were significantly associated with suicidal ideation (OR = 1.14, 95% CI 1.02–1.27, *p* = 0.021) (Table 5).

ROC curve analysis demonstrated that serum S100B levels showed moderate discriminative ability for identifying patients with MDD and suicidal ideation. The area under the curve (AUC) was 0.74 (95% CI: 0.63–0.85, *p* = 0.002). The optimal cut-off value for serum S100B was 9.85 pg/mL, which yielded a sensitivity of 72.4% and a specificity of 69.0% (Table 6).

Regression analysis results for the mediation model examining the relationship between suicide probability, childhood trauma, and serum S100B levels are shown in Table 7. Suicide probability was a significant predictor of childhood trauma (B = 0.365, SE = 0.065, t = 5.632, *p* = 0.001). Suicide probability was also significantly associated with serum S100B levels (B = −0.272, SE = 0.035, t = −7.752, *p* = 0.001). In addition, childhood trauma was a significant predictor of serum S100B levels (B = −0.210, SE = 0.077, t = −2.709, *p* = 0.009) (Table 7).

The indirect effect of suicide probability on serum S100B levels through childhood trauma was additionally evaluated using bias-corrected bootstrapping with 5000 resamples. The bootstrapped indirect effect remained statistically significant because the 95% confidence interval did not include zero, supporting the presence of an indirect statistical association between suicide probability, childhood trauma, and S100B levels. The mediation analysis results of the indirect effect using Sobel, Aroian, and Goodman tests are shown in Table 8. The Sobel test demonstrated a statistically significant indirect effect (Z = −2.45, standard error = 0.031, *p* = 0.014). Consistent with this finding, the Aroian test also indicated a significant indirect effect (Z = −2.43, standard error = 0.032, *p* = 0.015). Similarly, the Goodman test showed a statistically significant indirect effect (Z = −2.48, standard error = 0.030, *p* = 0.013) (Table 8).

**Figure 1:**
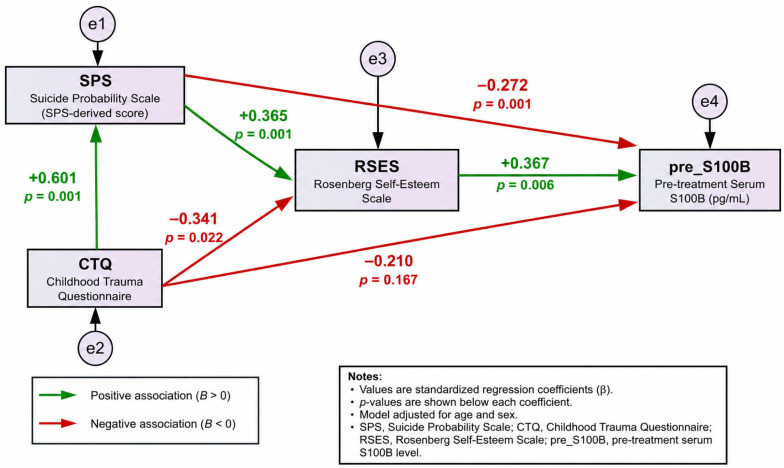
Exploratory path analysis showing the associations among SPS-derived suicide probability scores, childhood trauma, self-esteem, and pre-treatment serum S100B levels in patients with MDD and suicidal ideation. Positive and negative regression coefficients are explicitly indicated.

Figure 1 presents the path analysis examining the relationships among SPS-derived suicide probability scores, childhood trauma, self-esteem, and pre-treatment serum S100B levels in patients with MDD and clinically defined suicidal ideation. Suicide probability was positively associated with childhood trauma (B = 0.365, *p* = 0.001) and negatively associated with pre-treatment serum S100B levels (B = −0.272, *p* = 0.001). Childhood trauma also showed a significant negative association with serum S100B levels (B = −0.210, *p* = 0.009). The path between self-esteem and serum S100B levels was not statistically significant (*p* > 0.05) (Figure 1).

**Figure 2:**
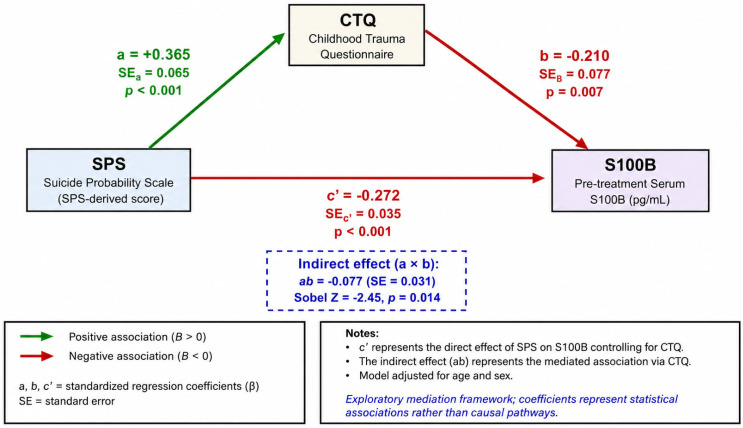
Exploratory mediation model illustrating the indirect statistical association between SPS-derived suicide probability scores and pre-treatment serum S100B levels through childhood trauma. Standardized regression coefficients and coefficient directions are displayed.

Figure 2 presents the mediation model evaluating the indirect effect of childhood trauma on the relationship between suicide probability and pre-treatment serum S100B levels. Suicide probability was significantly associated with childhood trauma (B = 0.365, *p* = 0.001), and childhood trauma was significantly associated with serum S100B levels (B = −0.210, *p* = 0.009). The indirect effect of suicide probability on serum S100B levels through childhood trauma was statistically significant, as demonstrated by the Sobel test (Z = −2.45, *p* = 0.014), Aroian test (Z = −2.43, *p* = 0.015), and Goodman test (Z = −2.48, *p* = 0.013) (Figure 2).

**Figure 3:**
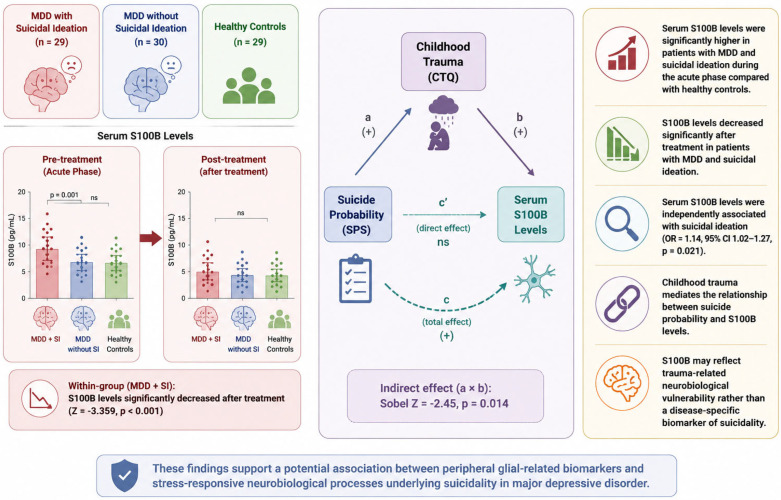
Summary of the main findings of the study. Serum S100B levels were elevated in patients with major depressive disorder and suicidal ideation during the acute phase and decreased after treatment. Childhood trauma showed an indirect statistical association in the relationship between suicide probability and serum S100B levels, suggesting that S100B may reflect trauma-related neurobiological vulnerability rather than a disease-specific biomarker of suicidality. ns: Non significant.

Figure 3 summarizes the main findings of the study, highlighting elevated serum S100B levels in patients with MDD and suicidal ideation during the acute phase, the reduction in S100B levels after treatment, and the indirect statistical association between suicide probability and S100B levels through childhood trauma (Figure 3).

## 3. Discussion

In the current study, serum S100B levels were significantly higher in patients with MDD with suicidal ideation compared with healthy controls before treatment, whereas this difference was no longer observed after treatment. In addition, mediation analyses demonstrated that the relationship between suicide probability and serum S100B levels was not direct but occurred indirectly through childhood trauma. These findings suggest that S100B may represent a state-dependent biological marker associated with active depressive episodes accompanied by suicidal ideation and that early-life trauma may play a key mediating role in this association.

### 3.1. S100B Levels in MDD and Changes with Treatment

Elevated peripheral S100B levels in patients with MDD have been reported in several previous studies. Arolt et al. showed that serum S100B concentrations were higher in patients with major depression compared with controls and suggested a possible relationship with treatment response [28]. Ambrée et al. reported that S100B levels could predict treatment response in patients with melancholic depression, supporting the idea that S100B reflects disease activity rather than a stable trait [24]. Our findings are consistent with these reports, as S100B levels were elevated during the acute phase of illness and decreased following treatment, reaching levels comparable to those of healthy controls.

Meta-analytic evidence further supports the association between S100B and depressive symptom severity. Tural et al. demonstrated a positive correlation between serum S100B levels and the severity of depression in patients with MDD [20]. Although depression severity was not directly modeled in our analyses, the normalization of S100B levels after treatment indirectly supports the view that S100B may track clinical improvement. However, the present findings should be interpreted cautiously because treatment was administered in a naturalistic clinical setting without standardized therapeutic protocols. Consequently, the observed reduction in S100B levels may reflect multiple overlapping factors, including antidepressant exposure, hospitalization-related stabilization, reduction in physiological stress, or general improvement in depressive psychopathology, rather than changes specifically related to suicidality.

### 3.2. Suicidal Ideation and S100B

The relationship between suicidality and S100B has attracted increasing attention. Falcone et al. reported that serum S100B levels were associated with suicidality in adolescent psychiatric inpatients, suggesting that S100B could be a potential biomarker of suicide risk [29]. Dogan et al. found higher postmortem cerebrospinal fluid S100B levels in individuals who died by suicide compared with non-suicidal deaths [25]. While these findings suggest a link between S100B and suicidal behavior, our results add an important nuance: the association between suicide probability and S100B levels was not direct but mediated by childhood trauma. This indicates that S100B may not be a specific marker of suicidal ideation per se, but rather a biological correlate of underlying vulnerability factors that increase suicide risk.

Interestingly, the regression analysis in the present study revealed a negative association between suicide probability and serum S100B levels (B = −0.272). This finding differs from several previous studies reporting positive correlations between S100B levels and suicidality. For example, Falcone et al. reported elevated S100B levels in adolescents with suicidal ideation and suggested that S100B may represent a biological marker associated with suicide risk [29]. Several factors may explain this discrepancy. First, differences in study populations may play an important role. Falcone et al. investigated adolescent psychiatric inpatients, whereas the present study included adult patients with major depressive disorder. Age-related differences in astroglial activation and neuroinflammatory responses may influence the relationship between S100B levels and suicidality. Second, our analytical approach incorporated childhood trauma as a mediating factor. The mediation analysis suggests that the relationship between suicide probability and S100B levels may operate indirectly through trauma-related neurobiological mechanisms rather than representing a direct association. This interpretation is consistent with the hypothesis that S100B may reflect trauma-related neurobiological vulnerability rather than functioning as a direct suicide-related risk process. More precisely, peripheral S100B may represent a non-specific stress-responsive biomarker associated with glial signaling and neurovascular stress physiology rather than a direct biological marker of suicidality itself. Nevertheless, this interpretation should be approached cautiously given the relatively modest sample size of the present study. Although the study was sufficiently powered to detect large between-group differences in serum S100B levels, mediation and multivariable regression analyses generally require substantially larger samples for stable estimation of indirect effects and pathway coefficients. Therefore, the observed mediation pattern may be vulnerable to statistical instability, suppression effects, or reduced reproducibility, and should be regarded as exploratory rather than confirmatory evidence.

Although the group-level analysis demonstrated elevated serum S100B levels in patients with MDD and suicidal ideation, the regression-based mediation model yielded negative coefficients for the associations of suicide probability and childhood trauma with S100B levels. This apparent discrepancy is important and should be interpreted cautiously. One possible explanation is a suppression effect arising from the simultaneous inclusion of highly interrelated psychosocial variables in a relatively small sample. Alternatively, differences in variable scaling, residual confounding, or model instability may have influenced the direction of the regression coefficients. Therefore, the mediation model should not be interpreted as demonstrating a causal or biologically definitive pathway. Instead, it represents an exploratory statistical association suggesting that the relationship among suicide probability, childhood trauma, and S100B may be more complex than a simple linear biomarker model.

### 3.3. Childhood Trauma as a Mediating Factor

Previous studies by Falcone et al. demonstrated associations between elevated S100B levels, suicidality, and childhood trauma in adolescent psychiatric populations [27,29]. However, these studies primarily reported cross-sectional associations and did not investigate the mechanistic pathways linking trauma exposure, suicide risk, and S100B levels. In contrast, the present study extends this literature by applying a formal mediation framework and demonstrating that childhood trauma statistically mediates the relationship between suicide probability and S100B levels in adult patients with major depressive disorder.

Childhood trauma is a well-established risk factor for both MDD and suicidal behavior. Falcone et al. demonstrated an association between childhood trauma exposure and serum S100B levels in adolescent psychiatric inpatients, proposing that early-life stress may induce long-lasting neurobiological alterations reflected in glial markers [27]. Our findings extend this literature to an adult MDD population, showing that childhood trauma (as measured by the CTQ) mediated the relationship between suicide probability and serum S100B levels. This suggests that early adverse experiences may contribute to alterations in glial function or neuroinflammatory pathways, which are subsequently reflected in peripheral S100B levels and associated with increased suicide risk.

Self-esteem has also been proposed as an important psychological mediator in the relationship between childhood trauma and suicidal ideation. Duprey et al. reported that self-esteem partially mediated the association between childhood maltreatment and suicidal ideation in emerging adults [8]. In contrast, in our study, self-esteem did not show a significant direct association with serum S100B levels. This discrepancy may reflect differences in study populations, outcome measures, or the fact that S100B represents a biological rather than a psychological pathway linking trauma to suicidality. In addition, the mediation findings should not be interpreted as evidence of a definitive mechanistic pathway. Given the limited sample size and observational design, the indirect associations identified in the present study are better viewed as hypothesis-generating statistical relationships that require replication in larger longitudinal cohorts. Small-sample mediation models are particularly susceptible to coefficient instability and variability in indirect effect estimation.

Furthermore, the indirect effect was additionally supported by bias-corrected bootstrapped confidence intervals, which are considered more robust than classical Sobel-type methods in mediation analysis. Nevertheless, given the modest sample size and observational design, these findings should still be interpreted as exploratory rather than confirmatory evidence.

### 3.4. Potential Biological Mechanisms

S100B is predominantly produced by astrocytes and is involved in neuroplasticity, neuroinflammation, and blood–brain barrier (BBB) integrity. Janigro et al. emphasized that peripheral S100B levels should not be interpreted solely as markers of neuronal damage, but rather as indicators of complex glial and BBB-related processes [18]. Gayger-Dias et al. further highlighted that S100B can cross brain barriers and that peripheral levels may be influenced by both central and extracerebral sources [17]. In this context, the elevated S100B levels observed during the acute phase of MDD with suicidal ideation may reflect increased glial activation or BBB permeability associated with stress and inflammation, whereas normalization after treatment may indicate restoration of neurobiological homeostasis. Importantly, peripheral S100B should not be interpreted as a disease-specific marker of neuronal injury or suicidality. Contemporary evidence suggests that circulating S100B levels may reflect a broader biological stress-response system involving astroglial signaling, endothelial dysfunction, blood–brain barrier permeability changes, and systemic inflammatory activation. Moreover, extracerebral sources—including adipocytes, melanocytes, and peripheral immune-related tissues—may also contribute to serum S100B concentrations under conditions of physiological or psychological stress. Therefore, peripheral S100B likely represents a non-specific neurovascular and stress-responsive biomarker rather than a direct indicator of a single neuropsychiatric mechanism.

It is also important to note that S100B lacks disease specificity. Elevated peripheral S100B levels have been reported in a range of neurological and systemic conditions, including traumatic brain injury, neurodegenerative disorders, and intense physical stress. Consequently, S100B should be interpreted as a non-specific marker of glial activation or blood–brain barrier alterations rather than a disorder-specific biomarker.

Childhood trauma may influence peripheral S100B concentrations through chronic stress-related neurobiological processes involving hypothalamic–pituitary–adrenal (HPA) axis dysregulation, low-grade systemic inflammation, endothelial dysfunction, and altered neurovascular signaling. Persistent exposure to early-life stress may contribute to astroglial activation and subtle blood–brain barrier alterations, potentially facilitating the peripheral release of glial-associated proteins such as S100B. However, given the multifactorial determinants of circulating S100B levels, these mechanisms remain speculative and should not be interpreted as evidence of a specific causal pathway linking childhood trauma to suicidality.

### 3.5. Clinical Implications

From a clinical perspective, the present findings should be interpreted cautiously. Serum S100B is not a disease-specific biomarker and may be elevated in a variety of neurological and systemic conditions. Therefore, S100B cannot be considered a standalone diagnostic marker for suicidal ideation or major depressive disorder. Instead, it may provide complementary biological information when interpreted alongside clinical and psychosocial assessments. In this context, peripheral S100B may provide complementary information regarding stress-related neurovascular and glial signaling processes rather than functioning as a direct or disease-specific biomarker of suicide risk.

The present study contributes to the existing literature in several ways. Unlike previous studies, we evaluated longitudinal changes in S100B levels before and after treatment in patients with active suicidal ideation. In addition, we integrated psychosocial and biological variables within a mediation framework, demonstrating that childhood trauma mediates the association between suicide probability and S100B levels. These findings suggest that S100B may reflect trauma-related neurobiological vulnerability rather than serving as a direct biomarker of suicidality. Future longitudinal studies incorporating repeated biomarker measurements in both suicidal and non-suicidal MDD populations will be necessary to determine whether S100B changes are specifically associated with suicidality or instead reflect broader treatment-related biological changes in depressive illness.

### 3.6. Strengths and Limitations

The strengths of this study include well-matched patient and control groups, the assessment of key psychosocial variables such as childhood trauma and self-esteem, and the use of both regression-based mediation analysis and multiple tests (Sobel, Aroian, and Goodman) to confirm the significance of indirect effects.

Nevertheless, several limitations should be acknowledged. First, the relatively modest sample size represents an important limitation, particularly for multivariable regression and mediation analyses. Although the a priori sample size estimation suggested adequate power for detecting large between-group differences in serum S100B levels, the study was not specifically powered for stable estimation of indirect effects or multivariate pathway models. Mediation analyses conducted in relatively small samples may be especially vulnerable to coefficient instability, suppression effects, model overfitting, and reduced reproducibility. Consequently, the indirect associations identified in the present study should be interpreted cautiously as exploratory and hypothesis-generating findings rather than definitive evidence of a stable mechanistic pathway. Replication in larger adequately powered longitudinal studies is necessary. Second, the naturalistic clinical design introduced substantial heterogeneity and potential confounding factors. Several clinically relevant variables—including illness duration, recurrence status, depressive subtype, prior treatment history, and pharmacological treatment regimens—were not systematically controlled or stratified. Because antidepressants, antipsychotic augmentation, hospitalization-related stabilization, and general clinical recovery may independently influence inflammatory and neuroglial biomarkers, the observed reduction in serum S100B levels cannot be confidently attributed specifically to improvement in suicidality. Therefore, the longitudinal biomarker findings should be interpreted cautiously. Third, S100B levels were measured only at admission and discharge, and longer-term follow-up measurements (e.g., 1–6 months) were not available. Consequently, the long-term stability of S100B levels and their potential prognostic value could not be evaluated. Future studies with longitudinal designs and repeated biomarker assessments are needed to clarify the temporal dynamics of S100B in relation to depressive symptoms and suicidality. Fourth, although mediation analysis was used to evaluate statistical relationships among suicide probability, childhood trauma, and S100B levels, the observational nature of the data limits causal interpretation. The direction of paths in the mediation model reflects statistical associations rather than temporal relationships between variables. Fifth, psychological scales were administered only to patient groups, and comparable psychometric data were not obtained from healthy controls. Although these instruments primarily assess clinical risk factors in psychiatric populations, the absence of these measures in the control group limits the ability to compare psychological profiles across groups. Repeated biomarker measurements were not available in the non-suicidal MDD group, limiting direct longitudinal comparison between depressive subgroups.

Moreover, the mediation analysis was based on observational and largely cross-sectional data; therefore, temporal ordering and causality cannot be inferred. The negative direction of some regression coefficients despite elevated group-level S100B levels further indicates that the mediation findings may be affected by suppression or model specification issues. These results should therefore be considered hypothesis-generating and require confirmation in larger longitudinal studies. Although bootstrapped confidence intervals were used to improve the robustness of mediation analyses, the relatively small sample size may still limit the stability and reproducibility of indirect effect estimates. Finally, although patients with MDD without suicidal ideation were included to support the mediation analysis of psychosocial variables, S100B levels were not longitudinally assessed in this group. Therefore, the present findings cannot determine whether elevated S100B levels are SPS-derived suicide probability or reflect depressive pathology more broadly. Prospective studies incorporating multiple patient groups, larger samples, and comprehensive biomarker panels—including markers of neuroinflammation and blood–brain barrier integrity—would help clarify the biological pathways linking childhood trauma, depressive pathology, and suicidality. This asymmetry in longitudinal biomarker assessment substantially limits interpretation of treatment-related S100B dynamics. Specifically, the present design cannot distinguish whether the observed decrease in S100B levels reflects suicidality-specific biological changes or more general effects of depression treatment and clinical recovery.

## 4. Materials and Methods

### 4.1. Study Design and Setting

This study used a mixed observational design combining cross-sectional case–control comparisons with a longitudinal repeated-measure biomarker assessment. Baseline comparisons were performed among patients with MDD and suicidal ideation, patients with MDD without suicidal ideation, and healthy controls, whereas repeated pre- and post-treatment serum S100B measurements were obtained only in the suicidal ideation group. It was conducted at the Department of Psychiatry, Gaziantep University Faculty of Medicine, Turkey, between October 2019 and September 2020. The primary objective was to evaluate serum S100B levels in patients with major depressive disorder (MDD) and suicidal ideation before and after inpatient treatment and to investigate the associations between S100B levels, suicide probability, childhood trauma, and self-esteem. The study protocol was approved by the Gaziantep University Clinical Research Ethics Committee (Approval No: 2019/390, Date: 1 April 2019). All procedures were performed in accordance with the Declaration of Helsinki, and written informed consent was obtained from all participants prior to enrollment.

### 4.2. Patient Selection

A total of 89 individuals were enrolled in the study and categorized into three groups: patients with major depressive disorder (MDD) and suicidal ideation (n = 29), patients with MDD without suicidal ideation (n = 30), and healthy control participants (n = 29). Patients were consecutively recruited from individuals hospitalized in the psychiatry clinic with a diagnosis of MDD.

Inclusion criteria for the patient groups were a DSM-5 diagnosis of major depressive disorder, age between 18 and 65 years, voluntary participation with the ability to provide informed consent, absence of pharmacological treatment at admission, and completion of inpatient treatment with remission, defined as a Hamilton Depression Rating Scale (HDRS) score below 7 at discharge. For the suicidal ideation group, the presence of active suicidal ideation accompanied by a suicide plan was additionally required.

Exclusion criteria included refusal to participate; history of traumatic brain injury or cerebrovascular disease; neurodegenerative disorders or dementia; epilepsy or seizure history; current or previous electroconvulsive therapy; pregnancy; chronic systemic disease; alcohol or substance use disorder; schizophrenia or other psychotic disorders; smoking; and insufficient educational level to complete psychometric assessments.

The mean duration of hospitalization for patients with suicidal ideation was 14.4 days, and all patients in this group were discharged in remission. Healthy controls were recruited from the same geographical region during the same time period and had no history of psychiatric or neurological disorders, suicidal ideation, or suicide attempts.

Patients with MDD without suicidal ideation were included primarily to strengthen the mediation analysis examining the relationships among suicide probability, childhood trauma, and serum S100B levels. Because longitudinal S100B measurements were not obtained in this group, direct biomarker comparisons across all three groups were not the primary focus of the study. In this group, serum S100B concentrations were measured only once at baseline before treatment initiation, whereas longitudinal pre- and post-treatment measurements were obtained only in patients with MDD and suicidal ideation.

### 4.3. Clinical Assessment Procedure

All participants underwent a face-to-face psychiatric interview conducted by experienced psychiatrists. Diagnoses and psychiatric comorbidities were assessed using the Structured Clinical Interview for DSM-5 Disorders (SCID-5). Patients with any psychiatric comorbidity other than MDD were excluded. Sociodemographic data were recorded using a standardized data collection form. Psychometric assessments were administered to both patient groups prior to initiation of treatment. The control group did not complete psychometric scales.

### 4.4. Psychometric Instruments

Suicide Probability Scale (SPS): The Suicide Probability Scale is a 36-item, 4-point Likert-type self-report instrument designed to assess suicide risk in adolescents and adults. It evaluates four domains: hopelessness, suicidal ideation, hostility, and negative self-evaluation. Total scores range from 36 to 144, with higher scores indicating greater suicide risk. The Turkish version of the SPS has demonstrated high reliability and validity, with a Cronbach’s alpha coefficient of 0.89 [30]. Rather than measuring suicidal ideation alone, the SPS assesses a broader multidimensional construct of suicide-related risk and probability, including hopelessness, hostility, negative self-evaluation, and suicide ideation-related features.

Rosenberg Self-Esteem Scale (RSES): Self-esteem was assessed using the Rosenberg Self-Esteem Scale. In this study, the first 10 items of the scale were used. Total scores range from 0 to 30, with lower scores reflecting lower self-esteem. The Turkish adaptation has demonstrated satisfactory validity and reliability [31].

Childhood Trauma Questionnaire (CTQ): The CTQ is a retrospective self-report instrument consisting of 28 items rated on a 5-point Likert scale. It assesses five types of childhood trauma: emotional abuse, physical abuse, sexual abuse, emotional neglect, and physical neglect. Higher total scores indicate greater trauma exposure. The Turkish version has been validated and shown to be reliable in clinical populations [32].

Hamilton Depression Rating Scale (HDRS): Depression severity was assessed using the 21-item HDRS. This clinician-rated scale evaluates depressive symptoms over the previous week. The Turkish version has demonstrated strong psychometric properties [33].

### 4.5. Serum S100B Measurement

After a 12 h overnight fast, 5 mL of venous blood was collected from the antecubital vein into tubes without anticoagulant. Samples were allowed to clot at room temperature for 20 min and then centrifuged at 4000× *g* for 10 min. Serum was aliquoted into Eppendorf tubes and stored at −80 °C until analysis. Prior to analysis, samples were brought to room temperature. Serum S100B concentrations were measured using a commercially available human S100B ELISA kit (Elabscience Biotechnology Inc., Houston, TX, USA; Catalog No: E-EL-H2327), according to the manufacturer’s instructions. The analytical sensitivity of the assay was 2.34 pg/mL. The intra-assay and inter-assay coefficients of variation were <8% and <10%, respectively. All serum samples were analyzed in duplicate under standardized laboratory conditions by personnel blinded to clinical group allocation. To minimize analytical variability, patient and control samples were processed within the same analytical batch whenever possible.

### 4.6. Statistical Analysis

Sample size estimation was performed using G*Power version 3.1. Assuming a large effect size (Cohen’s d = 0.80), a significance level of α = 0.05, and statistical power (1 − β) of 0.80, the minimum required sample size was calculated as 26 participants per group. To account for potential dropouts or missing data, the target sample size was increased by approximately 10%, resulting in 29 participants in the primary patient and control groups. Importantly, this sample size estimation was based on detecting between-group differences in serum S100B levels and was not specifically designed to provide sufficient power for multivariable regression or mediation analyses.

Statistical analyses were performed using SPSS version 26.0 (IBM Corp., Armonk, NY, USA) and AMOS version 21.0. The normality of continuous variables was assessed using the Shapiro–Wilk test. As serum S100B levels did not show a normal distribution, non-parametric statistical methods were applied. The Mann–Whitney U test was used to compare S100B levels between independent groups, while the Wilcoxon signed-rank test was employed to evaluate changes in S100B levels before and after treatment within the suicidal ideation group. Independent-sample t-tests and chi-square tests were used to compare sociodemographic and categorical variables, as appropriate. Multivariable logistic regression analysis was performed to evaluate the association between serum S100B levels and suicidal ideation status among patients with major depressive disorder. Suicidal ideation status (presence vs. absence) was entered as the dependent variable. Serum S100B level was included as the primary independent variable of interest, while childhood trauma (CTQ score), self-esteem (RSES score), age, and sex were entered as covariates to adjust for potential confounding effects. Mediation analyses were conducted using regression-based path analysis to evaluate whether childhood trauma (CTQ score) mediated the association between suicide probability (SPS score; independent variable) and serum S100B levels (dependent variable). Indirect effects were primarily evaluated using bias-corrected bootstrapped confidence intervals with 5000 resamples, consistent with contemporary recommendations for mediation analysis. Classical Baron and Kenny criteria and Sobel-type tests (Sobel, Aroian, and Goodman) were additionally reported as supplementary exploratory analyses rather than primary inferential methods [34,35]. In addition to the classical Baron and Kenny approach, the robustness of the mediation results was further evaluated using bootstrapped confidence intervals with 5000 resamples. In order to explore whether serum S100B levels differed across all study groups, an additional comparison including patients with MDD with suicidal ideation, patients with MDD without suicidal ideation, and healthy controls was performed using the Kruskal–Wallis test. When significant differences were detected, post hoc pairwise comparisons were conducted using Dunn–Bonferroni correction. A two-tailed *p* value < 0.05 was considered statistically significant.

## 5. Conclusions

In conclusion, serum S100B levels were elevated during the acute phase of MDD with suicidal ideation and decreased following treatment; however, the specificity of this longitudinal change to suicidality could not be determined within the present study design. The exploratory mediation analysis suggested a statistically significant indirect association involving childhood trauma; however, this finding should be interpreted cautiously because the observational design and directionally inconsistent regression coefficients preclude causal inference. These findings suggest that peripheral S100B may reflect broader stress-responsive neurovascular and glial signaling processes associated with chronic psychosocial stress exposure rather than serving as a specific biomarker of suicidality.

## Figures and Tables

**Table 1:** Sociodemographic characteristics of the study groups.

Variable	MDD + SI (n = 29)	MDD − SI (n = 30)	Healthy Controls (n = 29)	*p* Value
Gender, n (%)				0.801 ^a^
Male	13 (44.8)	14 (46.7)	14 (48.3)	
Female	16 (55.2)	16 (53.3)	15 (51.7)	
Age, years (mean ± SD)	38.86 ± 13.4	39.71 ± 12.8	38.03 ± 12.1	0.874 ^b^
Education, years (mean ± SD)	9.62 ± 3.46	9.94 ± 3.88	10.41 ± 4.54	0.691 ^b^
Marital status, n (%)				0.845 ^a^
Single	8 (27.5)	9 (30.0)	9 (31.0)	
Married	16 (55.2)	15 (50.0)	14 (48.3)	
Divorced	5 (17.2)	6 (20.0)	6 (20.7)	
Baseline HDRS score (mean ± SD)	28.4 ± 5.2	22.1 ± 4.8	—	<0.001 ^c^

MDD: major depressive disorder; SI: suicidal ideation; HDRS: Hamilton Depression Rating Scale; SD: standard deviation. ^a^ Chi-square test. ^b^ One-way ANOVA. ^c^ Independent-sample *t*-test between MDD groups.

**Table 2:** Comparison of serum S100B levels between patients with MDD and suicidal ideation and healthy controls before and after treatment.

S100B (pg/mL)	MDD with Suicidal Ideation (n = 29)	Healthy Controls (n = 29)	*p* Value
Pre-treatment			
Median (Q1–Q3)	10.95 (9.08–28.95)	8.97 (7.11–10.00)	0.001 ^a^
Post-treatment			
Median (Q1–Q3)	7.84 (6.49–9.81)	8.97 (7.11–10.00)	0.323 ^a^
Within-group comparison			
Pre- vs. post-treatment	Z = −3.359	—	<0.001 ^b^

Q1, first quartile; Q3, third quartile; MDD, major depressive disorder. ^a^: Mann–Whitney U test. ^b^: Wilcoxon signed-rank test.

**Table 3:** Comparison of baseline serum S100B levels across the three study groups.

Group	n	Median S100B (pg/mL)	Q1–Q3
MDD with suicidal ideation	29	10.95	9.08–28.95
MDD without suicidal ideation	30	9.48	8.14–11.26
Healthy controls	29	8.97	7.11–10.00
Comparison	*p* value		
MDD + SI vs. Controls	0.012		
MDD + SI vs. MDD − SI	0.041		
MDD − SI vs. Controls	0.284		

MDD: major depressive disorder; SI: suicidal ideation. Overall group differences were evaluated using the Kruskal–Wallis test, followed by Dunn–Bonferroni post hoc comparisons.

**Table 4:** Comparison of clinical scale scores between patients with MDD with and without suicidal ideation.

Scale	MDD with Suicidal Ideation (n = 29)	MDD Without Suicidal Ideation (n = 30)	*p* Value
Suicide Probability Scale (SPS)	87.45 ± 11.14	46.20 ± 17.05	0.001 ^a^
Rosenberg Self-Esteem Scale (RSES)	14.28 ± 5.71	13.76 ± 5.34	0.725 ^a^
Childhood Trauma Questionnaire (CTQ)	45.17 ± 14.76	30.50 ± 12.41	0.001 ^a^

^a^: Independent-sample *t*-test.

**Table 5:** Logistic regression analysis predicting suicidal ideation.

Variable	OR	95% CI	*p*
S100B	1.14	1.02–1.27	0.021
Childhood Trauma (CTQ)	1.08	1.03–1.13	0.002
Self-esteem (RSES)	0.97	0.90–1.04	0.381
Age	1.01	0.98–1.04	0.462
Sex (female)	1.19	0.54–2.63	0.667

**Table 6:** ROC analysis of serum S100B levels for identifying MDD patients with suicidal ideation.

Parameter	AUC	95% CI	Cut-off	Sensitivity	Specificity	*p*
Serum S100B	0.74	0.63–0.85	9.85 pg/mL	72.4%	69.0%	0.002

**Table 7:** Regression analysis results for the mediation model examining the relationship between suicide probability, childhood trauma, and serum S100B levels.

Dependent Variable	Independent Variable	B	Std. Error	t Value	*p* Value
CTQ	SPS	0.365	0.065	5.632	0.001 *
S100B	SPS	−0.272	0.035	−7.752	0.001 *
S100B	CTQ	−0.210	0.077	−2.709	0.009 *

SPS: Suicide Probability Scale; CTQ: Childhood Trauma Questionnaire; S100B: serum S100B protein; B: unstandardized regression coefficient. * Statistically significant at *p* < 0.05.

**Table 8:** The mediation analysis results of the indirect effect using Sobel, Aroian, and Goodman tests.

Test	Z Value	Standard Error	*p* Value
Sobel test	−2.45	0.031	0.014
Aroian test	−2.43	0.032	0.015
Goodman test	−2.48	0.030	0.013

## Data Availability

The original contributions presented in this study are included in the article. Further inquiries can be directed to the corresponding author.

## Abbreviations

BBB	Blood–brain barrier
CTQ	Childhood Trauma Questionnaire
GFAP	Glial fibrillary acidic protein
MDD	Major depressive disorder
RSES	Rosenberg Self-Esteem Scale
S100B	S100 calcium-binding protein B
SD	Standard deviation
SE	Standard error
SPS	Suicide Probability Scale
